# Optimizing peritoneal dialysis catheter placement

**DOI:** 10.3389/fneph.2023.1056574

**Published:** 2023-04-11

**Authors:** Sana F. Khan, Mitchell H. Rosner

**Affiliations:** Division of Nephrology, University of Virginia Health System, Charlottesville, VA, United States

**Keywords:** dialysis (ESKD), peritoneal, dialysis catheter complications, outcomes - health care, long term optimal planning

## Abstract

Long-term success of peritoneal dialysis as a kidney replacement therapy requires a well-functioning peritoneal dialysis catheter. With ongoing reductions in infectious complications, there is an increased emphasis on the impact of catheter-related and mechanical complications. There is currently a marked variation in the utilization of various types of catheters (double cuff vs single cuff, coiled tip vs straight tip), methods of catheter insertion (advanced laparoscopic, open surgical dissection, image guided percutaneous, blind percutaneous), timing of catheter insertion, location of catheter placement (pre-sternal v. abdominal) and peri-operative practices. Specialized approaches to catheter placement in clinical practice include use of extended catheters and embedded catheters. Marked variations in patient lifestyle preferences and comorbidities, specifically in high acuity patient populations (polycystic kidney disease, obesity, cirrhosis) necessitate individualized approaches to catheter placement and care. Current consensus guidelines recommend local procedural expertise, consideration of patient characteristics and appropriate resources to support catheter placement and long-term functioning. This review focuses on an overview of approaches to catheter placement with emphasis on a patient-centered approach.

## Introduction: current placement of PD catheters and issues

It is reported that approximately 424,000 patients worldwide utilize peritoneal dialysis (PD) as a method of kidney replacement therapy (1). A well- functioning PD access is essential to PD technique success, empowering patients to perform a therapy associated with reduced cost and increased patient autonomy (2, 3). There is currently marked heterogeneity in the types of PD catheter used, insertion techniques, location of placement, timing of catheter insertion and peri-operative practices. Commonly reported complications include catheter flow restriction, exit site leaks, pain and infections with resulting termination of PD, delays or interruptions in treatment, emergency department visits or hospitalizations, and need for corrective procedures. Studies have reported 13-17% of PD technique failure are due to mechanical catheter complications (4–6). Additionally, there is variability in the definitions, reporting methods, choice of outcomes, and analysis of PD access outcomes which hampers the determination of best practices for PD catheter placement (7).

## What is optimal PD catheter placement: patient-centered approach

Knowledge of best practices in catheter insertion can minimize the risk of catheter complications leading to early PD failure. Guideline committees under the sponsorship of the International Society for Peritoneal Dialysis (ISPD) recommend optimal PD catheter implantation be based on individualized patient factors, facility resources and operator expertise (8). Currently, most PD catheters for chronic use are made of silicone rubber. There is a marked variability in the types of PD catheters available for use, and include the most common type of double cuff, straight or coiled-tip Tenckhoff catheters with a pre-formed arc bend in the intercuff segment. While single cuff catheters are available, double cuff catheter are thought to be superior in preventing peritonitis caused by periluminal entry of organisms (especially given variable compliance with prophylactic antibiotic ointment used to lessen the risk of exit site infections), and for firm tissue fixation of the catheter (9). There is no significant difference in functionality between straight- and coiled-tip catheters, with or without preformed arc bend (10–12). While coiled-tip catheters are theorized to have less incidence of inflow discomfort and better dialysate dispersion, these effects have not been studied specifically, hence catheter selection is determined largely by availability of local inventory.

Recent PD catheter registries have reported a marked variation in PD catheter insertion techniques that include open surgical dissection, laparoscopic insertion with advanced techniques, blind insertion *via* trocar and blind insertion *via* Seldinger technique (13, 14). An optimal patient centered approach balances local procedural expertise along with patient specific factors and local resource availability. A laparoscopic approach is utilized in patients generally considered safe for general anesthesia with a history of prior abdominal surgeries in addition to having flexibility of waiting for an operating room time (8). Moreover, the laparoscopic approach allows the performance of advanced surgical techniques including rectus sheath tunneling (which reduces risk of pericatheter leaks and prevents tip migration), prophylactic omentopexy and adhesiolysis (which can improve inflow and outflow of dialysate fluid). The ability to perform these proactive adjunctive techniques are thought to improve catheter outcomes (15–17). A recent metanalysis observed a significant reduction in flow obstruction and tip migration when advanced laparoscopic techniques were utilized (18). Additionally, the laparoscopic approach allows for concomitant repair of abdominal wall hernias. Percutaneous catheter insertions by radiologists or nephrologists have been utilized in patients needing PD access in an accelerated timeline (more timely than awaiting operating room time), no major prior abdominal surgeries, and it precludes the need for general anesthesia. However, the percutaneous technique obviates the ability to visualize adhesions, and perform advanced adjunctive techniques. An open surgical dissection method (under local or general anesthesia) has also been utilized for timely placement of PD catheters, both by nephrologists as well as surgeons. Similar to the percutaneous technique, advanced adjunctive techniques cannot be performed *via* open surgical technique (9, 19, 20).

Another tailored approach to optimizing outcomes is consideration of the timing of PD catheter insertion. There is a marked variation in estimated glomerular filtration rates (5- 8 ml/min) at the time of catheter placement (21, 22). Early planning and placement offers larger flexibility to resolve early insertion related problems. A well- organized urgent start PD initiation program with rapid access to catheter insertion allows for new dialysis patients to initiate and establish on PD rather than hemodialysis. However, a caution is that PD catheter use within 7 days of insertion has been showed to increase the risk of exit site leaks and infections, compared to standard initiation (28 days) (23). Specific catheter insertion related interventions to mitigate early complications include utilization of fibrin glue, and additional purse-string sutures at the level of deep cuff as well as near peritoneal membrane (24, 25).

Another strategy for timely initiation of PD is the embedded catheter technique, with the external limb of catheter embedded in subcutaneous tissue until the need for dialysis initiation. This allows for early patient commitment to PD, more predictable operating room scheduling, and immediate utilization of the catheter after exteriorization. However, there is a risk of non-usage of catheters, in addition to catheter dysfunction rates secondary to fibrin accumulation (26–28).

No specific catheter placement approach has been proven to produce superior outcomes. Comparison of percutaneous placement, open surgical dissection and basic surgical laparoscopy have shown equivalent patient outcomes (21, 29–31). However, studies investigating outcomes in advanced laparoscopic studies demonstrate superior outcomes compared to other approaches (18). Patient factors aside, optimal PD catheter placement involves operator expertise, and the ability to provide peritoneal access in a timely fashion. While seemingly simple, PD catheter placement is a critical life-sustaining procedure and patients benefit from experienced operators who are able to identify and rectify problems with catheter placement in a timely manner. When access to an operating room is rate-limiting, percutaneous insertion by a nephrologist for an appropriate patient is a reasonable option, and also provides ongoing continuity of care, patient satisfaction and high rates of peritoneal dialysis utilization (32).

## Approaches to PD catheter placement

**Figure 1:**
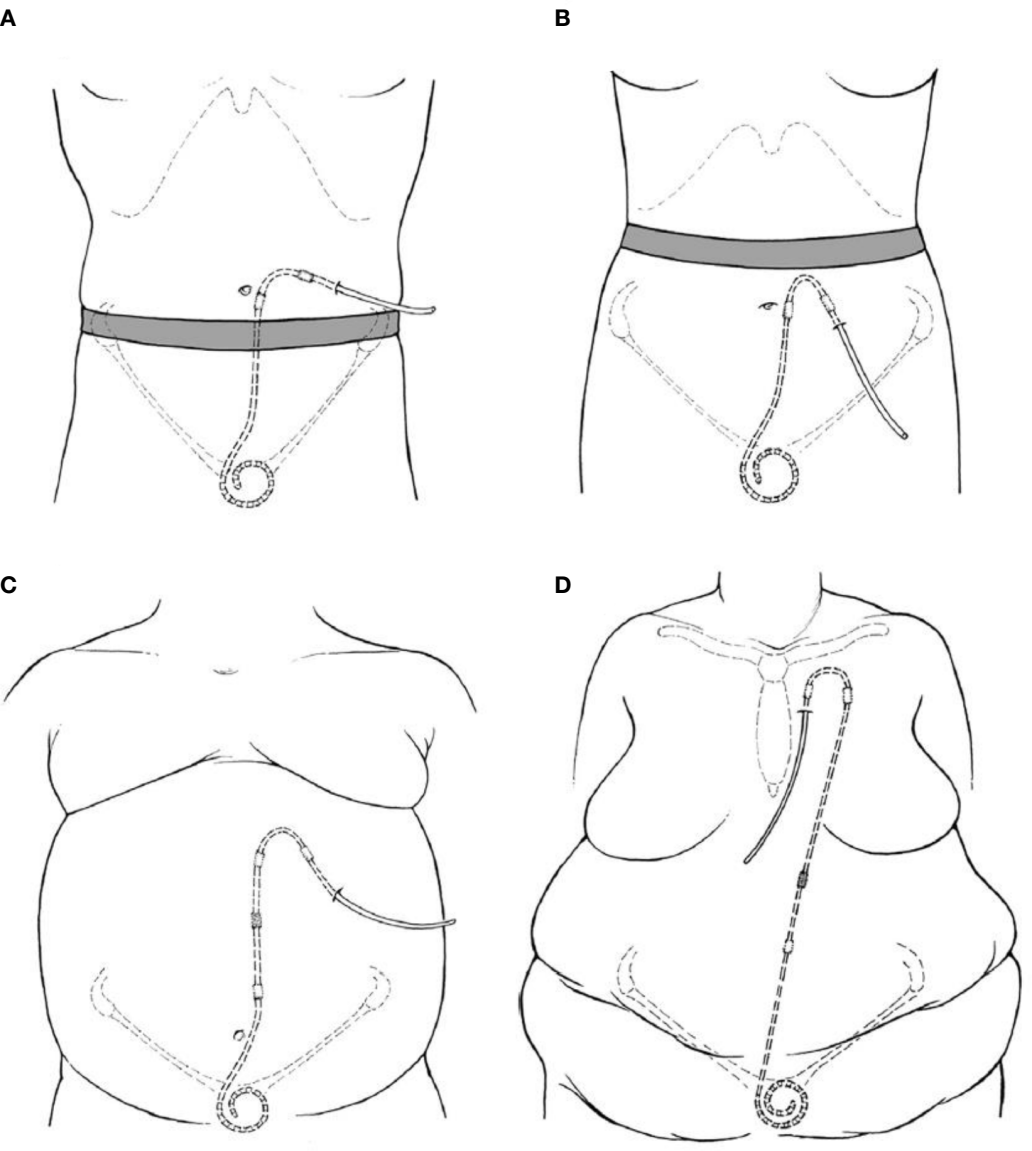
Illustrations of PD catheter positions with regards to exit site position **(A)** Laterally directed exit site emerging above the beltline. **(B)** Downward directed exit site below a high beltline. **(C)** Extended catheter system with an upper abdominal exit site. **(D)** Extended catheter system with a presternal exit site (9).

The goals of PD catheter placement involve a balance between pelvic position of catheter tip to facilitate inflow and outflow of dialysate along with an easily visible and accessible exit site. Operator expertise and experience necessitates selection of appropriate available catheter type suited to patient specific conditions. Placement includes the consideration of body habitus, belt-line, skin creases, prior scars, stomas, gastrostomy tubes, recreational habits and occupation. An ideal exit site is either above or below the beltline, with a lateral and inferior directed exit site (**Figures 1A, B**). However, based upon patient-specific criteria, other positions for the catheter exit site may be more appropriate. The catheter insertion site and length of intraperitoneal tubing determines the pelvic position of the catheter tip. While a catheter tip in the pelvis is preferred for optimal hydraulic function, excessively deep placement, or a catheter tip between the rectum and bladder can result in extrinsic catheter compression, flow impairment and pain with draining of the dialysate (33). In the pre-operative planning, the patient is examined while both supine and sitting, using the pubic symphysis as a guide to catheter tip location. Examination in the sitting position allows for visualization of an appropriate exit site (34).

Several patient factors necessitate utilization of an extended-length catheter system. Patients with excessive skin folds, stomas, incontinence, necessity for bath tub or other factors would benefit from an upper abdominal or pre-sternal exit site (**Figures 1C, D**). This can be achieved *via* a 2- piece extended catheter system, with a long subcutaneous section, while maintaining optimal catheter tip position. Alternatively, single piece catheters with long intercuff segments are also utilized in certain centers (35, 36).

Regardless of a standard vs extended approach, best practices in patient preparation catheter placement have been detailed in multiple publications and include appropriate selection of catheter type and exit site location, pre-op bowel preparation, utilization of a paramedian incision, deep cuff placement within or below the rectus muscle, lateral and downward directed exit site (8).

## Catheter placement in special populations

Several high acuity patient conditions (obesity, polycystic kidney disease, cirrhosis/ascites) necessitate a specialized approach for successfully conducting PD as a dialysis modality. This includes not only optimal exit site and catheter tip position, but utilization of focused interventions to ensure ideal long term function. Patients with obesity require creation of an easily visible upper abdomen/presternal exit site requiring an extended catheter approach. The catheter should be allowed to heal completely for several weeks prior to PD initiation. Ideally, a laparoscopic approach is recommended, utilizing selective omentopexy along with resection of epiploic appendices of sigmoid colon if needed. The upper abdominal and chest exit sites are located in areas with relatively thin subcutaneous layer, minimizing tubing stresses from mobility of the subcutaneous fat layer with postural changes. Data suggests longer time to first exit site infections in patients with extended catheters (37). Moreover, extended catheters enable peritoneal access in a subgroup of patients in whom conventional catheter placement would not be possible.

Patients with autosomal dominant polycystic kidney disease (ADPKD) present a unique set of concerns regarding limited peritoneal space, peritonitis risk and hernias. Several studies have reported the feasibility of PD in ADPKD patients (38–40). With regards to long term mechanical complications, these patients are at increased risk of abdominal wall hernias. The cause of these hernias is possibly multifactorial given increased intraperitoneal pressure in addition to possible collagen defects (41, 42). Simultaneous hernia repair and catheter insertion can safely be performed, and a tension- free hernia repair with prosthetic mesh is essential to minimize the risk of recurrence (43, 44).To prevent visceral injury to enlarged abdominal organs, laparoscopic ports, trocars and needles must be inserted and manipulated with increased caution. Ultrasound-guided percutaneous insertion of trocars is a feasible option for prevention of iatrogenic injury (45).

Patients with cirrhosis present their own unique challenges with regards to potential bacterial peritonitis, nutritional challenges, and concerns for leaks (46–49). Several centers have published their experiences regarding perioperative management of ascites. One center has reported catheter placement followed by 5-6 liters of large volume paracentesis. Thereafter, peritoneal drainage volume is allowed to exceed infused volume by 200 ml, allowing for a gradual and controlled drainage (47).Other centers have reported initiating low volume PD exchanges immediately following catheter placement. The drain volumes have been permitted to exceed infused volumes by 20% in certain reports, whereas others have reported an increase in 400-600 ml of effluent over the first few days. This allows for a safe and slow pattern of decompression, allowing for gradual ascites removal prior to training commencement (47–49).

## Conclusion

Safe and effective placement of PD access is critical to the ultimate outcomes of PD as a home dialysis modality. **Table 1** provides a guideline of potential PD catheter placement measures that home programs should consider monitoring to ensure continuous quality improvement. Ultimately, an effective PD catheter can serve the patient for many years and provide a life-sustaining therapy but this relies on an experienced team that carefully assesses each patient, identifies patient-specific issues that may impede optimal outcomes and addresses these issue through careful planning and monitoring.

**Table 1:** Factors to consider monitoring to ensure optimal pd catheter function and longevity.

Factor	Comments
• Identify patient-specific factors that may impeded catheter function or longevity: o Underlying medical conditions such as diabetes, obesity, ADPKD, cirrhosis/ascites o Hygienic issues o Specific activities that may jeopardize the catheter	• Utilization of patient-specific factors will determine: o Catheter placement technique and particular choice of catheters o Timing of catheter placement (urgent start, training period anticipated, potential issues for delayed wound healing) o Location of catheter exit site o Use of extended catheters o Specific training countermeasures to address risk of catheter malfunction
• Assess early catheter inflow and outflow of dialysate	• Dialysate should readily flow into and out of the peritoneal cavity with minimal to no pain
• Assess position of the PD catheter tip	• Catheter tip should in the lower, deep pelvis. This ensures optimal hydraulic function of the catheter and minimizes the risk for omental entrapment
• Assess position and integrity of exit site	• Location on the abdominal wall (away from belt-line, skin creases and folds • Should be easily visible by the patient • Direction of catheter exit: downward, lateral, or upward pointing • Location of superficial cuff in relation to exit site • Consideration of any risks for impaired wound healing or heightened risk of infections (immunocompromised)

NA, Not Applicable; Quant, Quantitative; Qual, Qualitative.

## Author contributions

SK and MR were responsible for preparation, writing and editing of this manuscript. Both authors contributed to the article and approved the submitted version.
